# Gut microbiome composition differs between aortic stenosis and aortic regurgitation patients

**DOI:** 10.1038/s41598-026-63354-w

**Published:** 2026-07-23

**Authors:** Benedikt Bartsch, Svetozar Nesic, Muntadher Al Zaidi, Raul Nicolas Jamin, Ansgar Ackerschott, Nikola Lübbering, Hannah Billig, Axel Schott, Chiara Hesse, Marijo Parcina, Marwan Hamiko, Farhad Bakhtiary, Georg Nickenig, Christian Kurts, Sebastian Zimmer, Christina Katharina Weisheit

**Affiliations:** 1 Department of Medicine-II, Heart Center Bonn, University Hospital Bonn Venusberg, Campus 1, 53127 Bonn, Germany; 2 Core Unit for Bioinformatics Data Analysis, Medical Faculty, University of Bonn, Venusberg-Campus 1, 53127 Bonn, Germany; 3 Institute of Medical Microbiology, Immunology and Parasitology (IMMIP), University Hospital Bonn, Venusberg-Campus 1, 53127 Bonn, Germany; 4 Department of Cardiac Surgery, Heart Center Bonn, University Hospital Bonn, Venusberg-Campus 1, 53127 Bonn, Germany; 5 Institute of Molecular Medicine and Experimental Immunology, Faculty of Medicine, University Hospital of Bonn University, Venusberg-Campus 1, 53127 Bonn, Germany; 6 Department of Anesthesiology and Intensive Care Medicine, University Hospital Bonn, Venusberg-Campus 1, 53127 Bonn, Germany; 7 Department of Anesthesiology and Intensive Care Medicine, University of Cologne, Cologne University Hospital, Kerpener Str. 62, 50931 Cologne, Germany

**Keywords:** Cardiology, Diseases, Gastroenterology, Medical research, Microbiology

## Abstract

The gut microbiome has emerged as an important modulator of cardiovascular diseases, yet data on aortic valve disorders, particularly aortic stenosis (AS), remain limited. This study aimed to characterize gut microbiome differences in patients with bicuspid (BS) and tricuspid aortic stenosis (TS) using aortic regurgitation (AR) patients as a clinically comparable control group with similar age distribution, comorbidities, and metabolic profiles. A total of 122 patients were included in this prospective cross-sectional study: 33 AR, 22 BS, and 67 TS patients. Microbiome profiling was conducted from anal swabs using 16S rRNA gene sequencing. Beta diversity was assessed via UniFrac distances, and computed redundancy analysis was performed using linear discriminant analysis effect size. The groups were largely homogeneous regarding most clinical characteristics; AR patients showed only marginally worse renal function, while BS patients were slightly younger with fewer cases of diabetes. In beta diversity analyses, both TS and BS patients exhibited clearly distinct microbiome compositions compared with AR controls, independent of clinical parameters used as potential confounders. TS and BS patients differed only minimally from each other. AR patients showed higher abundances of Bacteroides, Faecalibacterium, Lachnoclostridium, and Alistipes, whereas AS patients exhibited increased levels of Corynebacterium, Anaerococcus, Peptoniphilus, and Finegoldia. Co-abundance network analysis revealed that AS patients displayed an extensive and highly interconnected bacterial network, characterized by strong correlations among taxa such as Bacteroides, Alistipes, Parabacteroides, and Faecalibacterium, rather than isolated changes in individual taxa. AR patients provide a clinically suitable control group for AS. AS patients show a distinct microbiome composition and a highly interconnected microbial network with three major hubs, warranting further mechanistic investigation.

## Introduction

There is growing evidence linking gut microbiota to the development and progression of cardiovascular diseases, including aortic stenosis (AS). Tricuspid aortic valve stenosis is usually a disease of older adults driven by degenerative calcification of a previously normal three-cusp valve, often paralleling systemic atherosclerotic and inflammatory risk factors. Bicuspid aortic valve stenosis arises from a congenital two-cusp valve anatomy that creates abnormal shear stress, earlier leaflet fibrosis/calcification, and is frequently associated with aortopathy, including ascending aortic dilation^1^. Clinically, bicuspid stenosis tends to present decades earlier than tricuspid stenosis and requires closer assessment of the aortic root and ascending aorta^2^. The gut microbiome may contribute to both diseases by modulating systemic inflammation, lipid metabolism, mineralization pathways, and metabolites that could promote valvular endothelial dysfunction and calcific remodeling^3^.

The homeostasis of the gut microbiome is influenced by several factors, with diet, comorbidities, and pharmacotherapy, particularly antibiotic use, being the most significant determinants^4–7^. While alterations in gut microbiome composition are well documented in relation to cardiovascular risk factors such as obesity, diabetes, and hypertension, some studies suggest that AS is associated with a distinct microbial signature, despite overlapping comorbidities with other cardiovascular conditions (2, 10). In animal models, modulation of the gut microbiota through oral therapeutics has been shown to reduce systemic inflammation and slow the progression of cardiovascular diseases^4,7^. Recent clinical studies have supported these findings by highlighting the role of a pro-inflammatory gut-derived environment in promoting aortic valve calcification. Specifically, microbial metabolites such as lipopolysaccharides and trimethylamine N-oxide (TMAO) have been shown to amplify osteogenic signaling pathways involved in valvular calcification^8^. Together, these findings suggest that targeted modulation of the gut microbiome could offer a novel therapeutic avenue to influence the progression of AS.

However, data comparing microbiome composition across different aortic valve phenotypes, such as tricuspid versus bicuspid valve disease or aortic regurgitation and in patients consuming a Western diet remain scarce. Further research is needed to elucidate the potential of microbiome-targeted strategies in the prevention and treatment of aortic valve disease.

## Results

### Baseline characteristics

A total of 122 patients were categorized into three different groups: aortic regurgitation (AR, n = 33), bicuspid aortic stenosis (BS, n = 22) and tricuspid aortic stenosis (TS, n = 67). The clinical features of the enrolled patients are presented in Table 1. No significant differences were observed between the groups in terms of gender, smoking history, or metabolic parameters.

**Table 1:** Patient characteristics.

	Aortic regurgitation (n = 33)	Bicuspid AS (n = 22)	Tricuspid AS (n = 67)	P-value
Demographics				
Age (years)	67.3 ± 10	59.1 ± 10.9	65.8 ± 9.6	0.10
Gender (female)	7 (21.9%)	3 (13.6%)	16 (23.9%)	0.6
Admission physical examination				
Systolic BP, mmHg	131 ± 15	122 ± 18	129 ± 18	0.18
Diastolic BP, mmHg	70 ± 10	70 ± 10	71 ± 11	0.99
BMI, kg/m^2^	28.3 ± 3.3	27.8 ± 5.3	29 ± 4.9	0.54
Comorbidities				
CAD	12 (37.5%)	8 (36.4%)	37 (55.2%)	0.13
DM	5 (15.6%)	2 (9.1%)	24 (35.8%)	0.2
Smoking history	5 (15.6%)	4 (18.2%)	9 (13.4%)	0.82
TR	1 (3.1%)	0	6 (9%)	0.22
MR	4 (12.5%)	0	12 (17.9%)	0.12
Laboratory values				
eGFR (mL/min)	66.2 ± 17.9	78 ± 11.9	76.1 ± 15.3	0.06
BUN (mg/dL)	45 ± 20.9	35.6 ± 6.1	39.2 ± 19	0.14
Total Cholesterol (mg/dL)	158 ± 53	154 ± 42	158 ± 36	0.96
HDL (mg/dL)	44 ± 10	42 ± 8	48 ± 17	0.42
LDL (mg/dL)	88 ± 47	91 ± 35	90 ± 30	0.97
CRP (mg/L)	2.9 ± 2.9	4.8 ± 10.6	3.1 ± 4.1	0.4
WBC (G/L)	6.9 ± 1.7	7.4 ± 2.6	7.2 ± 1.8	0.66
Platelets (G/L)	217 ± 50	222 ± 43	220 ± 46	0.92
Hb (g/dL)	14.3 ± 1.8	14.8 ± 1.2	14.2 ± 1.3	0.21
HKT (%)	42.6 ± 5	43.1 ± 3.3	41.9 ± 3.4	0.43
CK-MB (U/L)	4.2 ± 7.2	2.96 ± 1.48	3 ± 1.7	0.34
Troponin (ng/L)	18.6 ± 16.9	13.6 ± 12.7	16.5 ± 12.5	0.44
Calcium (mmol/L)	2.38 ± 0.13	2.39 ± 0.17	2.38 ± 0.12	0.88
Phosphate (mmol/L)	1.24 ± 0.35	1.31 ± 0.35	1.28 ± 0.25	0.71
Total Bilirubin (mg/dL)	0.85 ± 0.51	0.83 ± 0.54	0.82 ± 0.56	0.97
ALT (U/L)	29 ± 14	32 ± 14	26 ± 14	0.24
AST (U/L)	29 ± 11	27 ± 10	27 ± 11	0.54
HbA1c%	5.7 ± 1.1	5.5 ± 0.49	5.9 ± 1	0.2
PT (sec)	25.1 ± 4	24.6 ± 2.4	24.1 ± 3.1	0.34
INR	1 ± 0.07	1 ± 0.07	1 ± 0.08	0.97

BP blood pressure, BMI body mass index, CAD coronary artery disease, DM Diabetes mellitus, TR severe tricuspid regurgitation, MR severe mitral regurgitation, GFR glormerular filtration rate, BUN blood urea nitrogen, HDL-c high-density lipoprotein cholesterol, LDL-c low-density lipoprotein cholesterol, ALT alanine aminotransferase, AST aspartate aminotransferase, PT prothrombin time, INR international normalized ratio. Values are presented as mean (± STD) or number (percent of group total). Analysis was performed using one-way ANOVA and Chi-square test.

### Alpha and beta diversity

Microbial diversity was analyzed within and across patient groups to investigate differences in gut microbiome composition. Alpha diversity describes the richness and evenness of taxa within individual samples, while beta diversity measures compositional variation between groups. Together, these metrics help determine whether disease states are linked to changes in overall microbial diversity or in specific taxonomic structures.

**Fig. 1:**
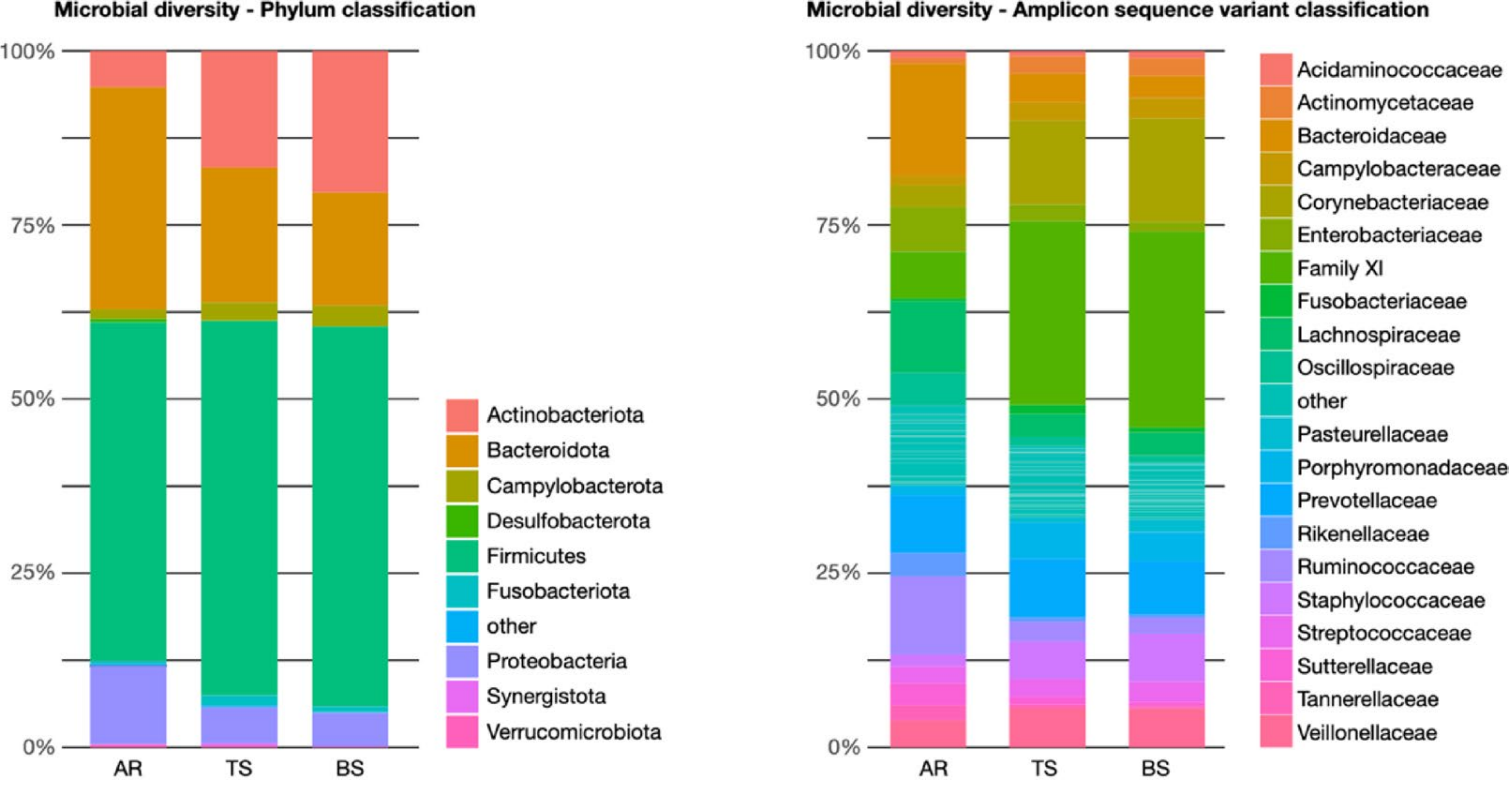
Microbiome composition Patients were categorized according to aortic regurgitation (AR), tricuspid aortic stenosis (TS), and bicuspid aortic stenosis (BS). Taxonomic classification of the microbiome samples was performed at the phylum level (**A**) and genus level (**B**).

There were no differences in alpha diversity among the three groups (Fig. 1). At the phylum level, the microbiome was mainly composed of Firmicutes, Bacteroidota, Actinobacteriota and Proteobacteria. AR patients showed higher abundance in Bacteroidota compared to BS and TS patients, while BS and TS patients showed increased levels of Actinobacteriota.

On amplicon sequence variant (ASV) level, the microbiome composition was primarily made up of Actinomycetaceae, Bacteroidaceae, Enterobacteriaceae and Prevotellaceae. Actinomycetaceae were overrepresented in AR, while Enterobacteriaceae were overrepresented in BS and TS.

**Fig. 2:**
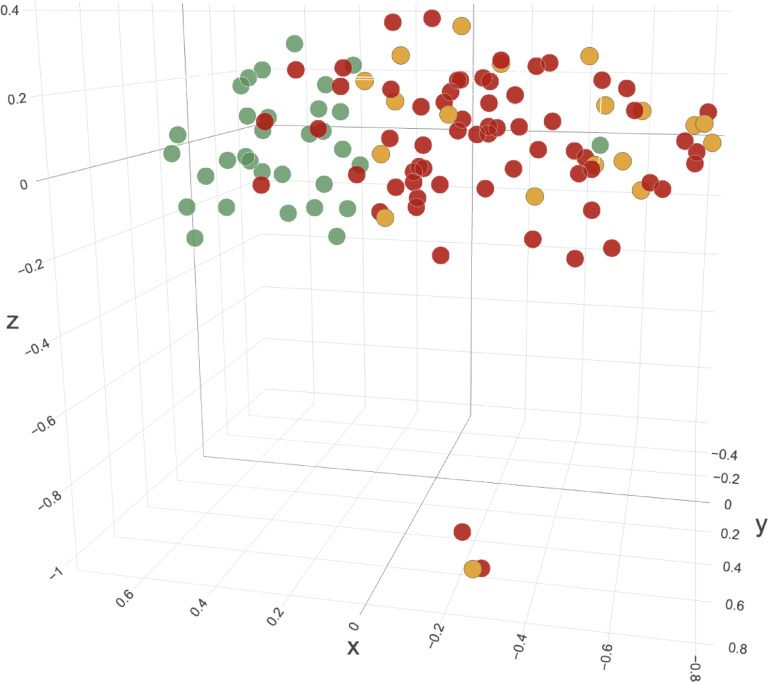
Unweighted UniFrac distance from microbiome samples.

To assess beta-diversity between patient populations, an unweighted UniFrac distance analysis was performed (Fig. 2). While both BS and TS groups were similar (R^2^ = 0.2; *p* = 0.18), there were significant differences between the AR and BS (R^2^ = 0.0015; *p* = 0.001), as well as the AR and TS groups (R^2^ = 0.0015; *p* = 0.001). PCoA analysis revealed little overlapping between AR and TS/BS microbiota (Fig. 2), while BS samples are broadly overlapping with other TS samples. Correspondingly, microbome composition seems to differ between patients with aortic stenosis and aortic regurgitation, irrespective of valve morphology.

Differences in microbiome composition between aortic regurgitation = green, bicuspid aortic stenosis = orange, and tricuspid aortic stenosis = red were calculated using the unweighted UniFrac distance and plotted graphically.

### Correlation of microbiome composition with age, gender and metabolic diseases

**Fig. 3:**
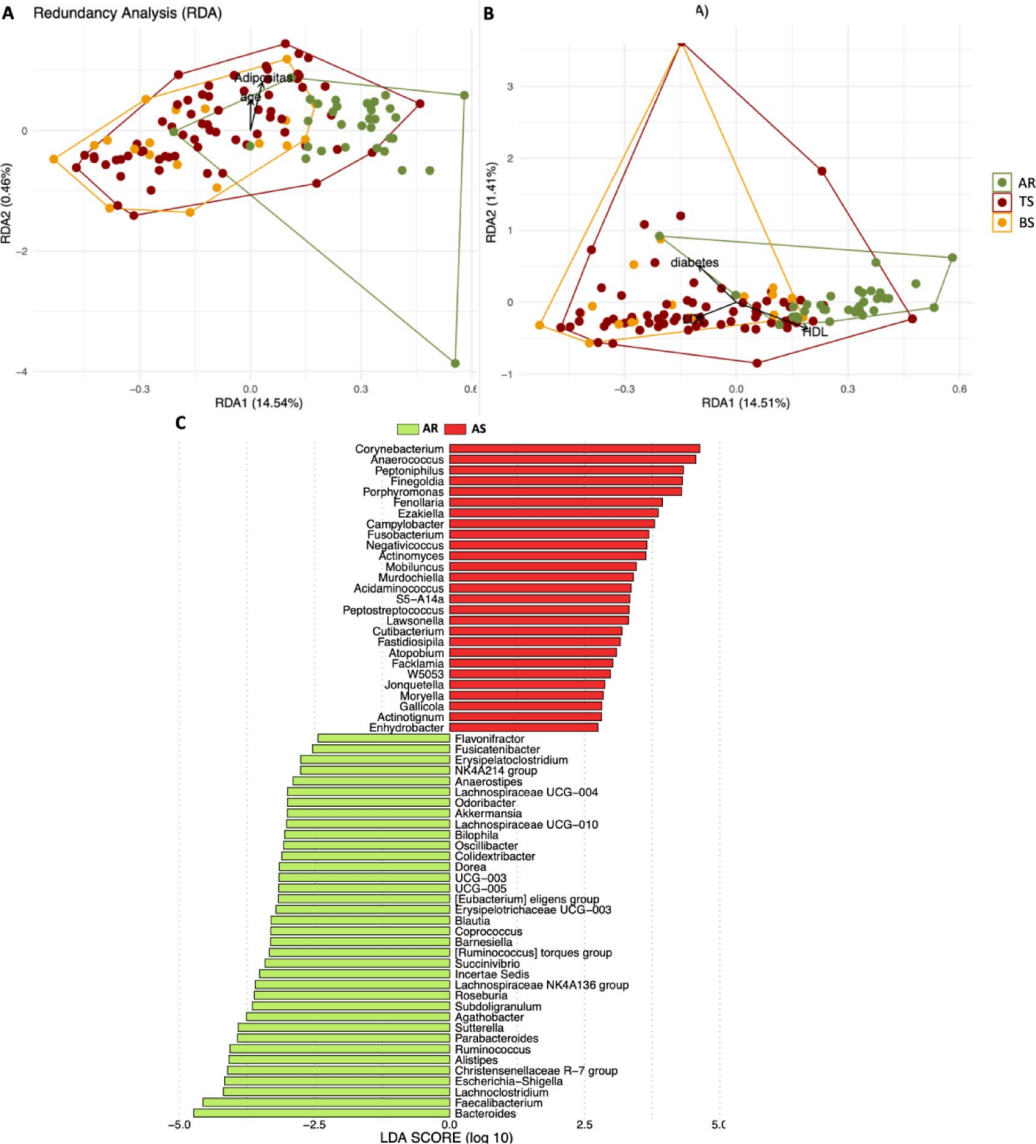
Computed redundancy analysis and linear discriminant analysis effect size. The influence of age, gender, and lipid disorders on the composition of the microbiome in the different treatment groups was analyzed using computed redundancy analysis (RDA) to identify potential confounding differences between the groups (**A**, **B**). Using Linear Discriminant Analysis Effect Size (LEfSe), 37 ASVs specific to AR and 28 ASVs specific to AS were identified in the microbiome (**C**). AR = aortic regurgitation, AS = aortic stenosis, BS = bicuspid aortic stenosis, TS = tricuspid aortic stenosis.

Age, gender and metabolic disorders (obesity, diabetes, dyslipidemia) are known to influence the gut microbiota. While no significant differences were detected between the groups with respect to age, gender or the occurrence of metabolic disorders (Table 1), the impact of these potential confounders on the differences in relative ASV abundance between groups was tested using computed redundancy analysis (RDA). RDA showed no correlation between diabetes, obesity, age, and only a weak correlation with blood lipid levels with ASV in BS, TS or AR (Fig. 3A-B).

### Gut microbiota differences between AR and AS patients

As TS and BS showed similar microbiome patterns when correlating with alpha and beta diversity, we combined them to search for the most discriminative ASVs between AR and AS using Linear Discriminant Analysis Effect Size (LEfSe) analysis. A total of 28 ASV were discriminative for AS and 37 ASV were discriminative for AR (Fig. 3C). Among these, the most common ones for AS were *Corynebacterium, Anaerococcus, Peptoniphilus* and *Finegoldia*, and for AR *Bacteroides, Faecalibacterium, Lachnoclostridium* and *Alistipes*.

### Analysis of co-abundance in AR and AS patients shows distinctly different microbial networks

Changes in the human microbiome, unless artificially altered, are rarely specific to a single bacterium but often involve entire clusters of bacteria that either increase or decrease in abundance. Therefore, a co-abundance analysis was performed at the ASV level to explore potential microbiome networks specific to AR or AS.

**Fig. 4:**
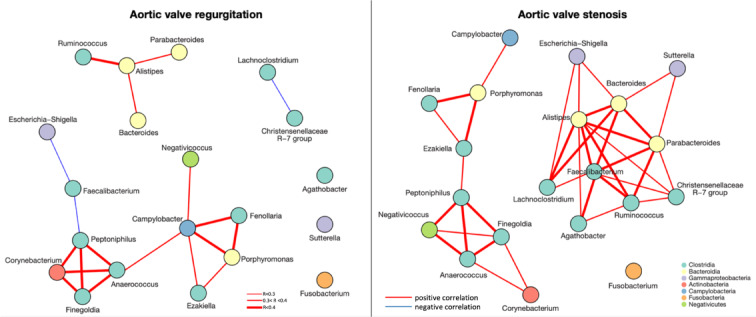
Co-abundance analysis in AR and AS patients Co-abundance analysis for AR (**A**) and AS (**B**) patients reveals two distinct clusters for each disease condition, with particularly strong correlations observed between clusters in AS patients. AR = aortic regurgitation, AS = aortic stenosis.

In AR patients, one network based around *Campylobacter* and *Anaerococcus* was distinctly correlated with four and three other taxa, respectively (Fig. 4A). In AS patients, two independent networks with positive correlations within each network were also observed (Fig. 4B). The first network consisted of *Peptoniphilus* and *Ezakiella*, each positively correlating with four other taxa. The second network was characterized by a closely interconnected cluster comprising *Bacteroides, Alistipes, Parabacteroides*, and *Faecalibacterium*. These taxa within the second network all correlate with each other, and each correlates with at least three other taxa within the cluster.

### Correlation between bacterial taxa and clinical features

To investigate the relationships between individual microbial taxa and clinical parameters, a Spearman correlation analysis with correction for multiple testing was performed between the gathered clinical findings (Table 1) and the 28 ASVs specific for AS and the 37 ASVs specific for AR. We identified four bacteria that correlated with individual laboratory values, regardless of group classification (AS/AR), with an effect size of at least R = 0.2. *Bacteroides* showed a positive correlation with troponin levels at admission (R = 0.2, *p* = 0.006). *Blautia* correlated with the liver enzyme alanine aminotransferase (ALT) (R = 0.2, *p* = 0.02). *Parabacteroides* (R = 0.3, *p* = 0.03) and *Subdoligranulum* (R = 0.4 *p* < 0.001) both exhibited a positive correlation with HDL levels at admission. Enterobacteriaceae abundance showed no significant association to TS or BS (ρ =  − 0.168, *p* = 0.119).

## Discussion

Our findings suggest that patients with different aortic valve diseases harbor distinct microbiome profiles. It may be speculated that the gut microbiome and aortic valve disease development are interconnected and this observation may provide new insights into pathophysiology and potentially, provide a preventive treatment target.

Bacterial richness, defined as the number of distinct bacterial taxa within a sample, is commonly used as a measure of gut microbiome diversity. Recent data suggest that microbial load is a major determinant of microbiome variation and thus a confounder in disease association. However, in our study, we did not find significant differences in microbiome richness between groups, thus limiting the impact of gut microbiome richness as a confounder^9^.

In contrast, microbiome composition in patients with TS and BS was significantly different from that of patients with AR in UniFrac analysis of beta diversity. However, differences between TS and BS in PCoA analysis were limited, despite the fact that BS patients tended to be younger and presenting with lower incidence of diabetes than TS and AR patients.

We did not observe a correlation between the microbiome composition of BS, TS or AR patients and lipid levels, age, diabetes or obesity, suggesting that the observed differences in microbiome composition could be related to the aortic disease rather than a result of comorbidities. These results differ from recently published data by Liu et al.^10^. However, the enrolled patients in that study consisted only of BS patients and were younger, with lower incidences of diabetes, BMI, and blood lipid levels^10^. Dietary differences between groups are also likely, given the variation in BMI as well as the ethnic and cultural differences between study populations.

On the phylum level, the microbiome of our cohort was comprised mainly of Actinobacteria, Bacteroidota, Desulfobacterota and Proteobacteria. However, Bacteroidota and Proteobacteria were more prominent in AR patients, while Actinobacteriota were abundant in TS and BS patients.

At the genus level AS patients showed an abundance of 28 ASVs specific to AS, the most abundant belonging to the genera *Corynebacterium, Anaerococcus, Peptoniphilus, Finegoldia* and *Fusobacterium*. Among these bacteria, several genera have already been linked to cardiovascular disease or its most prominent risk factors. *Peptoniphilus* species have been found in abundance in patients with coronary artery disease, and their reduction has been associated with decreased hypertension and glycemia^11,12^. *Fusobacterium*, especially *F. nucleatum*, induces macrophage-driven production of pro-inflammatory cytokines when present in the microbiome. This bacterium may enter aortic tissue and cause local inflammation as demonstrated in an atherosclerosis model^13,14^.

AR patients showed 37 discriminative ASV, the most common being *Bacteroides, Faecalibacterium, Lachnoclostridium, Escherichia-Shigella* and Christensenellaceae, *Parabacteroides, Sutterella, Blautia, Subdoligranulum*. Among these, *Bacteroides* has been shown to reduce intestinal lipopolysaccharide production, and its microbial enrichment has been associated with a reduction in atherosclerosis in a murine model^15,16^. These bacteria have also been shown to be overexpressed in microbial samples from patients with BS^10^. *Parabacteroides* has been extensively studied in atherosclerosis, and its enrichment in the murine microbiome has been associated with a reduction in atherosclerosis occurrence^7^. *Faecalibacterium* negatively correlated with the inflammatory marker hsCRP in a microbiome analysis of CAD patients^17^. However, microbiome analysis of Type II diabetes mellitus patients revealed increases in several *Faecalibacterium* species^18^. As diabetes mellitus was more frequent in AS patients—although not significantly increased—the overexpression of *Faecalibacterium* in the microbiome of AS patients may be due to the higher prevalence of diabetes in this group. Future studies are needed to disentangle this relationship.

*Lachnoclostridium* is associated with increased microbial trimethylamine-lyase activity, which promotes atherosclerosis^19^. *Escherichia-Shigella* remains poorly studied; however, it has been shown to correlate with heart failure^20^. Christensenellaceae, *Blautia* and *Subdoligranulum* have all been associated with lipid level variations, although a direct causal relationship has yet to be demonstrated^21–23^.

Over the past few years, it has been shown that microbiome alterations are not limited to individual bacteria but also involve the formation of symbiotic networks of various bacterial taxa, which may be either enriched or diminished within the microbiome^10,24^.

In this context, identifying key bacterial genera as target pathogens can be insightful, as they may serve as central hubs within a network, either promoting or preventing dysbiosis^10,25^. Co-abundance analysis revealed a small, loosely connected network centered around *Campylobacter* and *Anaerococcus* in AR patients, whereas AS patients exhibited a strong hub comprised of *Bacteroides, Parabacteroides, Alistipes*, and *Faecalibacterium*, which were highly correlated with each other and with other bacterial taxa. Notably, *Bacteroides, Parabacteroides*, and *Faecalibacterium* may act as key regulators, as they showed significant differences in abundance between AR and AS in our study, correlate with a variety of different bacteria and have been previously described in other studies as potential regulators of a reduced inflammatory response in cardiovascular diseases^7,15–17^.

In the future, it may be promising to analyze whether changes in the microbiome influence the progression of AS. In recent years, the metabolism of branched-chain amino acids and lipopolysaccharides has emerged as particularly relevant in cardiovascular research^7,16^.

There are several limitations to this study. Although patients with aortic regurgitation were included as a non-stenotic valvular disease comparison group, the absence of a healthy control cohort matched for age, sex, and cardiovascular risk factors limits our ability to determine whether the observed microbiome differences are specific to aortic stenosis or reflect broader disease-associated or baseline interindividual variation. Future studies should therefore include appropriately matched healthy controls to validate disease-specific microbiome alterations in aortic stenosis. Due to the different aortic pathologies compared in this study and their respective pathophysiology, a perfect matching of risk factors was not possible. Given our study design, we can only infer correlations, but not causal relationships. To better distinguish these phenomena, a targeted microbiome modification of the bacteria that differed significantly in our study could be performed in animal models.

Future studies could further explore whether modulating gut microbiota, particularly *Bacteroides*, *Parabacteroides*, and *Faecalibacterium*, could serve as a therapeutic strategy for AS. Additionally, longitudinal studies and interventional trials are needed to establish causal links between microbiome alterations and aortic valve disease progression.

In summary, our study is the first to demonstrate differences in microbiome composition between patients with AR and AS.

## Methods

### Ethics statement

Clinical investigations were performed in accordance with the Declaration of Helsinki. The study was reviewed and ethical approval was granted by the ethics committee of the University of Bonn, Germany (AZ Nr. 078/17). All subjects provided written informed consent before entering the study.

### Study population and sample collection

Rectal swab samples were collected from inpatients at the Heart Center Bonn, University Hospital Bonn from 2020 to 2023, and stored at − 80 °C prior to DNA extraction. All patients underwent heart surgery for AS or regurgitation (AR), with diagnoses confirmed via cardiac ultrasound. Exclusion criteria included patients with psychiatric or cognitive impairments that could interfere with providing informed consent, as well as those with acute infections, inflammatory bowel disease, other inflammatory conditions, gastrointestinal disorders, recent antibiotic or probiotic treatments within the past four weeks, active malignancies and autoimmune diseases. Participants were subsequently categorized based on their final clinical diagnosis.

### DNA isolation, 16S rRNA gene amplification, bioinformatics and statistical analysis

DNA extraction from stool samples was performed using the QIAamp DNA Fast Stool Mini Kit, automated on the QIAcube (Qiagen, Hilden, Germany). The samples were transferred to Power Bead tubes (Qiagen) containing 1.1 ml of InhibitEx lysis buffer. For swab samples, the QIAamp UCP Pathogen Mini Kit, also automated on the QIAcube, was used. The swabs were transferred into Pathogen Lysis Tubes S containing 0.65 ml of ATL buffer (with DX) and incubated at 56 °C for 10 min, shaking continuously at 600 rpm. Bead beating for both stool and swab samples was performed using the SpeedMill PLUS (Analytik Jena, Jena, Germany) for 45 s at 50 Hz, followed by the manufacturer’s protocol. Extracted DNA was stored at − 20 °C until PCR amplification. Blank extraction controls were included during the extraction process.

Amplicon sequencing of the variable regions 3 and 4 of the 16S rRNA gene was conducted as previously described in detail^26^.

### Statistical analyses of microbiota analyses

Clinical data are summarized as mean ± standard deviation (SD) or as counts with percentages, depending on the variable. Continuous variables among the three patient groups (AR, BS, TS) were compared using one-way analysis of variance (ANOVA), while categorical variables were analyzed with the Chi-square test.

Microbial alpha diversity was calculated at the amplicon sequence variant (ASV) level. Beta diversity differences between groups were measured using unweighted UniFrac distances and visualized through principal coordinate analysis (PCoA). Statistical significance of beta diversity variation between groups was tested with permutational multivariate analysis of variance (PERMANOVA).

Redundancy analysis (RDA) was performed using ASV-level data to explore the relationship of age, sex, obesity, diabetes, and lipid levels to microbiome composition.

Group-specific differences in microbial taxa were identified using linear discriminant analysis effect size (LEfSe), with the standard logarithmic LDA score applied to define discriminatory features.

Microbial community relationships were explored through a co-abundance network analysis at the ASV level, based on correlation coefficients between taxa abundances.

Correlations between individual bacterial taxa and clinical laboratory measurements were examined with Spearman’s rank correlation. Multiple testing correction was applied, and only associations with correlation coefficients of at least R ≥ 0.2 and adjusted *p*-values < 0.05 were considered statistically significant.

Some statistical analyses were conducted using R and QIIME2 packages, with a two-sided *p*-value < 0.05 regarded as significant.

Clinical characteristics of the study population are presented in Table 1. A total of 127 individuals were initially sampled. However, following quality control and the exclusion of low-quality samples (low input material), the final cohort consisted of 122 subjects.

## Data Availability

The data that support the findings of this study are available from the corresponding author, BB, upon reasonable request. The datasets generated and/or analysed during the current study are available in the European Nucleotide Archive (ENA) repository, under study accession number PRJEB109043 (https://www.ebi.ac.uk/ena/browser/view/PRJEB109043).
